# Role of PRY-1/Axin in heterochronic miRNA-mediated seam cell development

**DOI:** 10.1186/s12861-019-0197-5

**Published:** 2019-07-15

**Authors:** Avijit Mallick, Ayush Ranawade, Bhagwati P. Gupta

**Affiliations:** 1 Department of Biology, McMaster University, ON L8S-4K1, Hamilton, Canada; 2 Department of Physics, Harvard University, NW256, 52 Oxford St, Cambridge, MA 02138 USA

**Keywords:** *C. elegans*, *C. briggsae*, *Pry-1*, Axin, WNT asymmetry pathway, Seam cell, miRNA, Heterochronic development

## Abstract

**Background:**

*Caenorhabditis elegans* seam cells serve as a good model to understand how genes and signaling pathways interact to control asymmetric cell fates. The stage-specific pattern of seam cell division is coordinated by a genetic network that includes WNT asymmetry pathway components WRM-1, LIT-1, and POP-1, as well as heterochronic microRNAs (miRNAs) and their downstream targets. Mutations in *pry-1*, a negative regulator of WNT signaling that belongs to the Axin family, were shown to cause seam cell defects; however, the mechanism of PRY-1 action and its interactions with miRNAs remain unclear.

**Results:**

We found that *pry-1* mutants in *C. elegans* exhibit seam cell, cuticle, and alae defects. To examine this further, a miRNA transcriptome analysis was carried out, which showed that *let-7* (*miR-48, miR-84, miR-241*) and *lin-4* (*lin-4, miR-237*) family members were upregulated in the absence of *pry-1* function. Similar phenotypes and patterns of miRNA overexpression were also observed in *C. briggsae pry-1* mutants, a species that is closely related to *C. elegans*. RNA interference-mediated silencing of *wrm-1* and *lit-1* in the *C. elegans pry-1* mutants rescued the seam cell defect, whereas *pop-1* silencing enhanced the phenotype, suggesting that all three proteins are likely important for PRY-1 function in seam cells. We also found that these miRNAs were overexpressed in *pop-1* hypomorphic animals, suggesting that PRY-1 may be required for POP-1-mediated miRNA suppression. Analysis of the *let-7* and *lin-4*-family heterochronic targets, *lin-28* and *hbl-1*, showed that both genes were significantly downregulated in *pry-1* mutants, and furthermore, *lin-28* silencing reduced the number of seam cells in mutant animals.

**Conclusions:**

Our results show that PRY-1 plays a conserved role to maintain normal expression of heterochronic miRNAs in nematodes. Furthermore, we demonstrated that PRY-1 acts upstream of the WNT asymmetry pathway components WRM-1, LIT-1, and POP-1, and miRNA target genes in seam cell development.

**Electronic supplementary material:**

The online version of this article (10.1186/s12861-019-0197-5) contains supplementary material, which is available to authorized users.

## Background

**Fig. 1:**
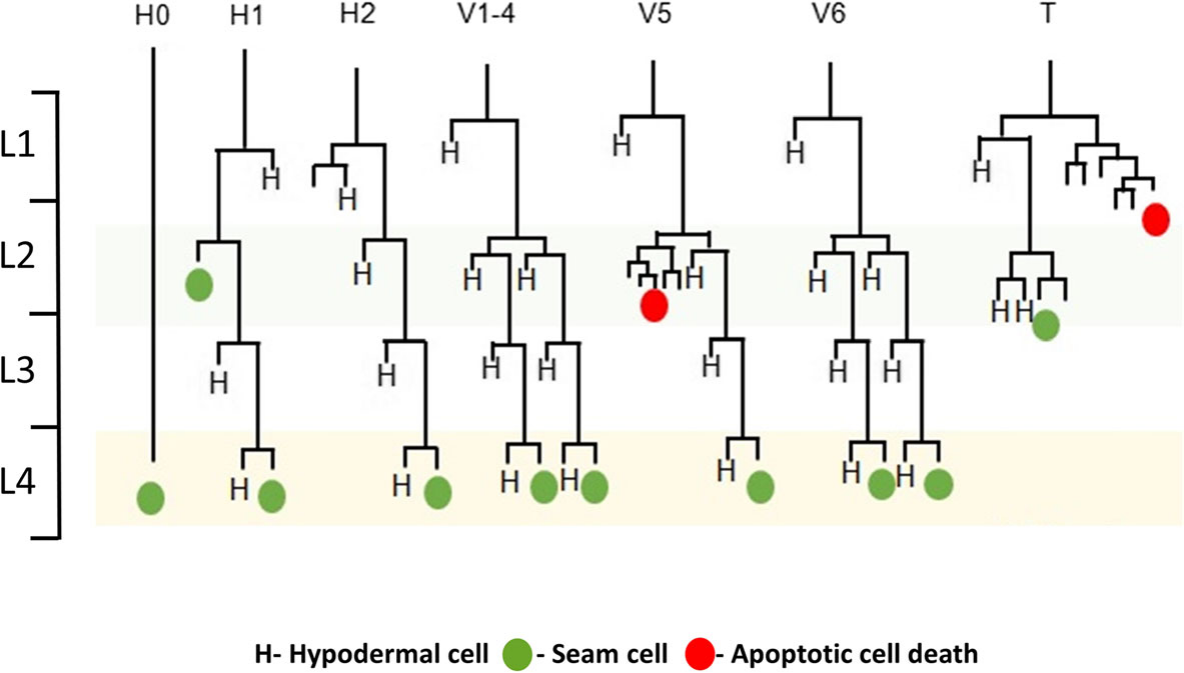
Seam cell asymmetric divisions during postembryonic development in *C. elegans*. During postembryonic development, *C. elegans* larvae undergo a series of molts, each of which is associated with carefully timed seam cell divisions. In the V1–4, and V6, cells undergo stem cell-like division where the anterior daughter fuses with the hyp7 syncytium. The posterior daughter then self-renews as another ‘stem cell-like’ seam cell. The exception to the asymmetric divisions is in early L2 stage where they undergo one symmetrical division prior to dividing again in an asymmetric manner

*Caenorhabditis elegans* hypodermal seam cells serve as a good model to elucidate spatiotemporal patterns of division and differentiation that enable cells to adopt sex-specific fates. They comprise two lateral rows of multipotent somatic cells which extend from the anterior to the posterior of the nematode (Fig. 1), and that divide in a stem cell-like manner to both self-renew, and generate daughter hypodermal, neuronal, and neuronal-support cells [1]. At the early L2 stage of development, the six seam cells (V1–6) undergo symmetric division to give rise to a total of 16 seam cells. By the end of the L4 stage, the cells terminally differentiate and fuse with their neighbors to give rise to adult cuticular structures called alae [2].

Multiple genes and pathways have been shown to regulate seam cell division and differentiation. These include the *lin-4* (*lin-4* and *miR-237*) and *let-7* (*let-7, miR-48, miR-84,* and *miR-241*) families of heterochronic microRNAs (miRNAs) that regulate the relative timing of developmental events [3–7]. Specifically, *lin-4* is required for the L1–L2 transition [2, 8], while the *let-7* family members act at later stages of development [9]. Mutations in *lin-4*, *miR-48, miR-84,* and *miR-241* cause cells to reiterate stages, which ultimately affects both the number of differentiated seam cells, and cuticle development [9].

Previous studies have shown that heterochronic miRNAs regulate a number of targets, including *hbl-1*, *lin-14*, and *lin-28* [10]. HBL-1 is a zinc-finger transcription factor that critically mediates embryogenesis [11], and that controls developmental timing during post-embryonic development [9]. LIN-14 is a novel class of transcription factor [12] that is initially expressed at high levels in hypodermal blast cells in newly hatched L1 animals but at lower levels by the L2 stage [13]. LIN-28 is a conserved RNA-binding protein that controls the maturation of *let-7* miRNA [5, 14–16]. Hypomorphic and null alleles of *lin-14* and *hbl-1* cause an increase whereas *lin-28* mutants cause a decrease in the overall number of seam cells [9, 17, 18].

In addition to heterochronic miRNAs and their targets, seam cell division is also regulated by the divergent WNT asymmetry pathway, whose components include the β-catenins WRM-1 and SYS-1, the NEMO-like kinase (NLK) LIT-1, and the T-cell factor/lymphoid enhancer factor (TCF/LEF) POP-1 [18, 19]. Removal of POP-1 activity causes seam cells to divide symmetrically, and thereby leads to an increase in their number. Conversely, since LIT-1 normally forms a complex with WRM-1 to phosphorylate and thus stimulate POP-1 export from the nucleus, disrupting WRM-1 and/or LIT-1 activity reduces the number of seam cells [19]. Similarly, the ratio of nuclear POP-1/SYS-1 activity affects the fate of daughter cells, such that those with lower POP-1 (and hence comparatively higher SYS-1) levels retain their seam cell fate, whereas those with higher POP-1 (and hence comparatively lower SYS-1) levels differentiate [20–23]. Genetic studies have also shown that WNT asymmetry pathway components interact with heterochronic genes to control seam cell development [17, 18].

While investigating the role of *pry-1* in developmental and post-developmental processes, we observed that *pry-1(mu38)* animals exhibit weaker cuticle and abnormal alae. Further analysis revealed that they also display a higher number of seam cells, a phenotype that was previously reported [19]. Similar defects were also seen in a *C. briggsae pry-1* mutant *Cbr-pry-1(sy5353)* [24], suggesting a conserved role for *pry-1* in seam cell development and cuticular alae formation. These observations are in line with our recent *pry-1(mu38)* mRNA transcriptome profiling (RNA-Seq), which identified differentially expressed (DE) genes associated with ‘cuticle development’ [25]. Given that the heterochronic pathway involves both protein-coding and miRNA genes, in the present study we conducted an miRNA-specific whole genome RNA-Seq experiment, which uncovered six DE miRNAs that include members of *lin-4* and *let-7* families. To understand the interaction of *pry-1* with miRNAs during seam cell development, we knocked-down WNT asymmetry pathway components. Reducing *wrm-1* and *lit-1* expression suppressed, while silencing *pop-1* exacerbated the *pry-1* phenotype. Furthermore, an miRNA expression analysis conducted in a *pop-1* hypomorph revealed a similar upregulation of miRNAs to that observed in *pry-1(mu38)* worms, suggesting both that POP-1 is critical for asymmetric seam cell division, and that its nuclear levels are likely reduced in *pry-1* mutants. Overall, our data demonstrates the importance of PRY-1 and its interactions with the WNT asymmetry pathway components for the regulation of miRNAs (and their targets) during asymmetric cell division. Since the WNT pathway and miRNAs are conserved in eukaryotes, similar interactions with Axin family members may control stem cell division in other systems.

## Results

**Fig. 2:**
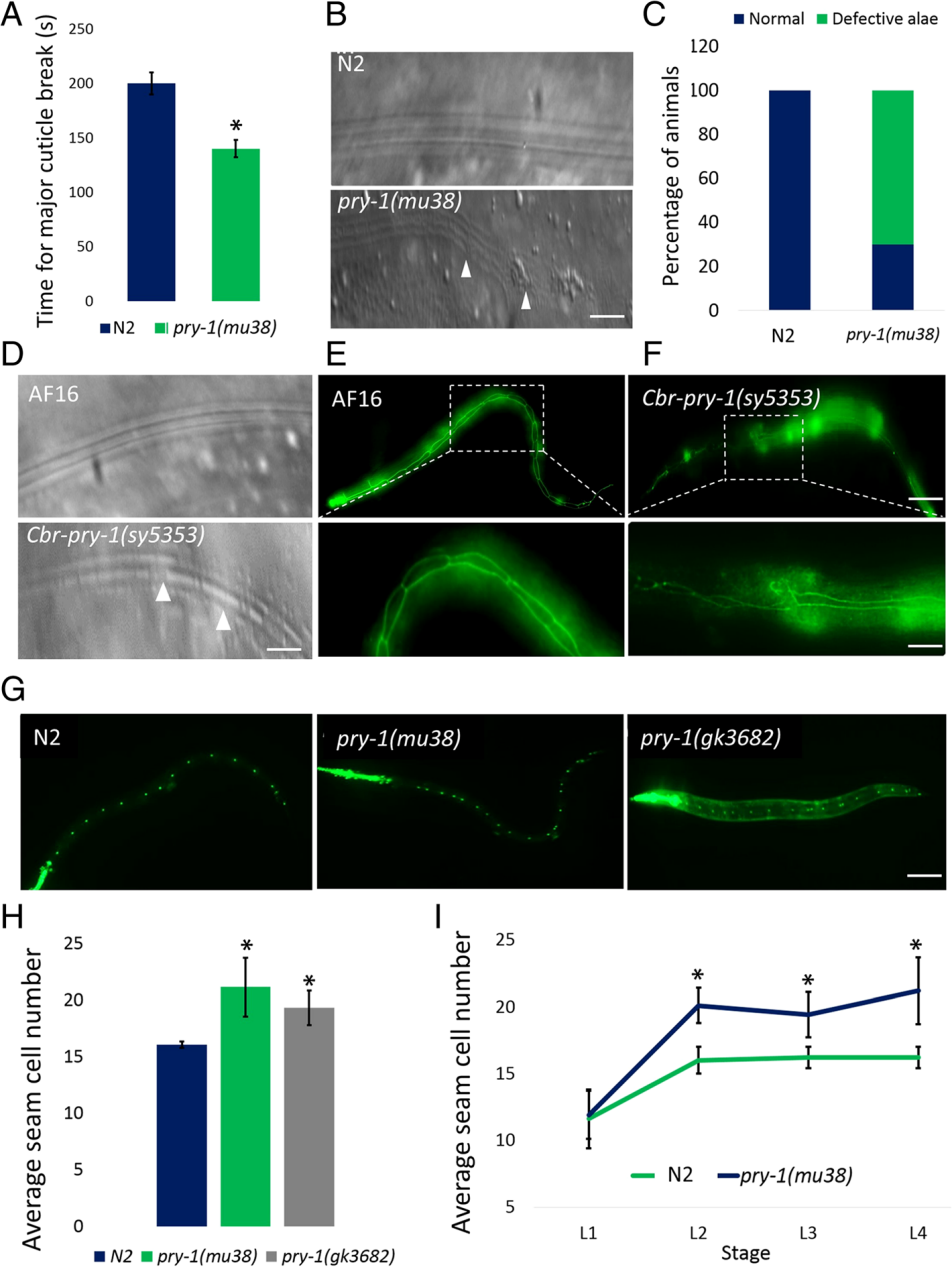
*pry-1* mutants display seam cell defects in *C. elegans* and *C. briggsae*. Mutations in *pry-1* lead to somatic defects. (**a**) Cuticle is weaker as demonstrated by the cuticle break assay. (**b, c**) *pry-1* mutants having defective alae formation. (**b**) Control N2 animals have distinct rows of alae. *pry-1(mu38)* mutants have defect with frequent breaks (see arrow). (**c**) Quantification of alae defect in control N2 and *pry-1(mu38)* animals. (**d**-**f**) Heterochronic phenotypes in *C. briggsae* AF16 and *Cbr-pry-1* mutants. (**d**) Alae defects are visible in *Cbr-pry-1(sy5353)*. (**e**, **f**) Seam cells in control AF16 (**e**) and *Cbr-pry-1(sy5353)* (**f**) are visualized by adherens-junction-associated marker *Cel-dlg-1p::GFP. Cbr-pry-1(sy5353)* animals show defects in cell fusion (scale bar 0.1 mm). Boxed areas, marked by dotted lines, have been enlarged in the second row. Scale bars in **b**, **d**-**f** are 0.01 mm. (**g**) Both the *pry-1(mu38)* and *pry-1(gk3682)* animals show increased seam cell numbers compared to control N2 (scale bar 0.1 mm). (**h**) Each control animal has exactly 16 seam cells, whereas an average of 21 and 19 cells are found in *pry-1(mu38)* and *pry-1(gk3682)* mutants, respectively. (**i**) *pry-1(mu38)* animals show increased seam cell number by the end of the L2 stage. In panels **a**, **h**, and **i**, each data point represents the mean of at least two replicates (each batch with 30 or more worms) and error bar represents the STD. Student’s *t*-test was used to determine the statistical significance: *p < 0.05

We observed that *pry-1* mutant worms have a weaker cuticle (Fig. 2a) and abnormal alae that frequently includes gaps (Fig. 2b, c). The phenotypic analysis of *C. briggsae pry-1* mutants, *Cbr-pry-1(sy5353)* [24], revealed similar gaps in alae as well as defective seam cell morphologies (Fig. 2d-f). Given these hypodermis-associated phenotypes, we investigated the role of *pry-1* in seam cell development.

### *pry-1* mutants exhibit an increased number of seam cells

In *C. elegans*, seam cells divide asymmetrically at each larval stage to produce two daughter cells, one of which fuses with the hypodermal syncytium, while the other retains the seam cell fate (Fig. 1). The L2 stage is unique because it also includes a symmetric division that causes an increase in the number of seam cells (Fig. 1). We found that the *pry-1(mu38)* mutants have an average of approximately five extra seam cells (Fig. 2g, h), consistent with a previously report [19]. A similar phenotype was also observed in *pry-1(gk3682)*, a new CRISPR/Cas9-induced mutant strain (provided by Don Moerman’s lab) (Fig. 2g, h; Additional file 1: Figure S1). A stage-specific analysis conducted using *scm::GFP* and *ajm-1::GFP* markers [26] revealed a higher number of seam cells in *pry-1* mutants by the end of the L2 stage, likely due to an increase in symmetric cell divisions (Fig. 2h, i). Together, these findings suggest that *pry-1* appears to play a role in L2-specific seam cell division.

### Heterochronic miRNA expression is altered in *pry-1* mutants

As described above, seam cell asymmetry is mediated by two interacting pathways. While heterochronic genes, such as members of the *lin-4* and *let-7* miRNA families, control cell division, the WNT asymmetry pathway plays a role in the specification of anterior/posterior daughter cell fates. To evaluate the involvement of miRNAs in *pry-1*-mediated seam cell development, we performed RNA-Seq experiment in L1-stage animals. The results revealed six DE miRNAs in the *pry-1(mu38)* mutants. Additionally, 61 novel miRNAs were recovered in the *C. elegans* reference sample (N2) (see Methods and Additional files 2-4: Table S1, S2, S3) that serve as a resource to further investigate the miRNA biology in worms.

**Fig. 3:**
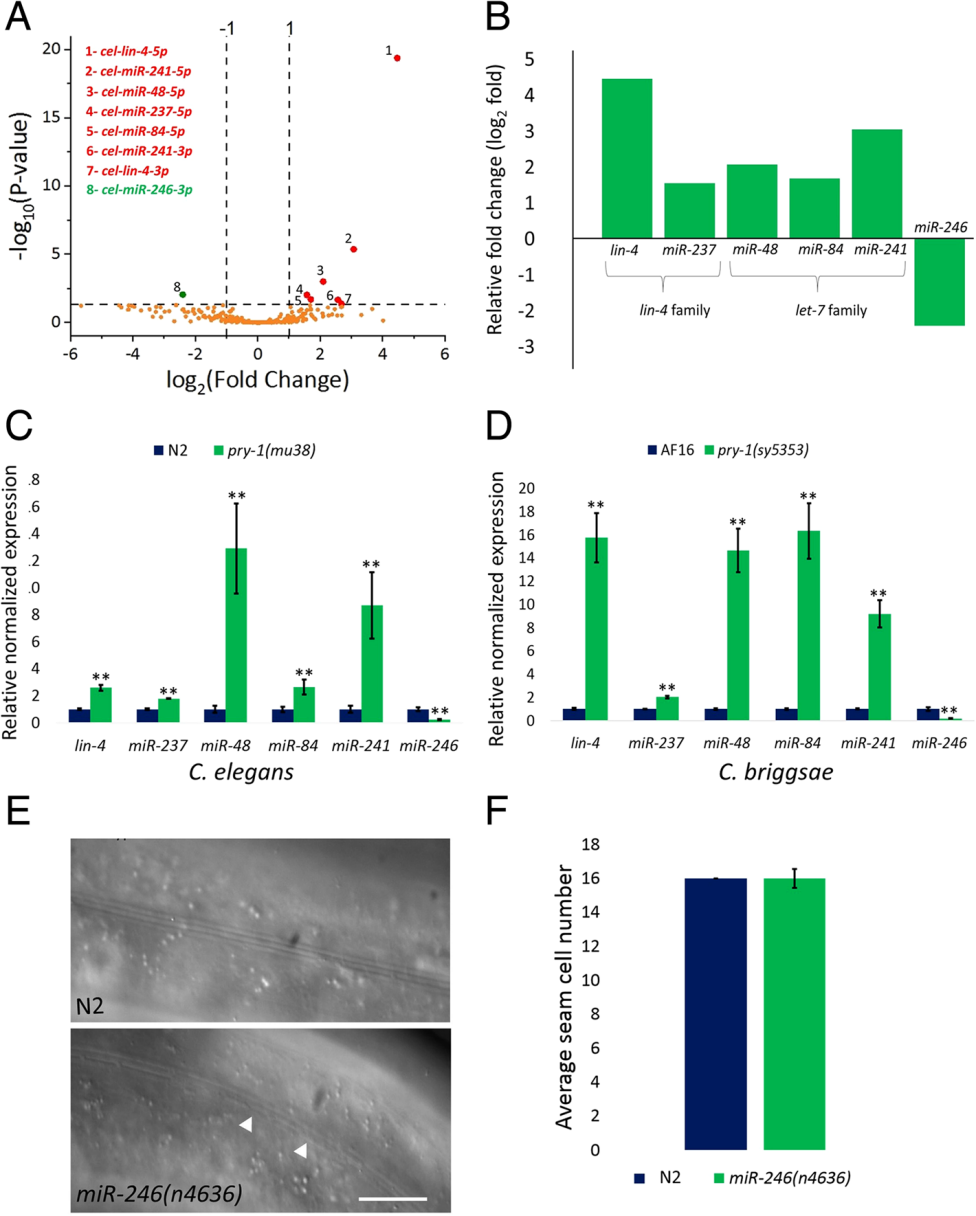
*pry-1* is necessary for normal miRNA expression in both *C. elegans* and *C. briggsae*. (**a**) Volcano plot of differentially expressed miRNA genes in *pry-1(mu38)*. Red and green dots mark significantly altered upregulated and downregulated transcripts, respectively, with a *p*-adj value of *<* 0.05. Orange dots mark transcripts that are not significantly altered (*p*-adj *>* 0.05). (**b**) Data showing log_2_ fold expression of miRNAs from RNA-Seq analysis. (**c**, **d**) qRT-PCR analysis of miRNAs in *Cel-pry-1(mu38) and Cbr-pry-1(sy5353)* animals at the L1 stage showed upregulation of all but *miR-246*. Each data point represents the mean of two replicates and error bar represents the SEM, **p < 0.01. (**e, f**) *miR-246(n4636)* mutants exhibit alae defect in the form of gaps (scale bar 0.01 mm) (**e**) but show no change in the number of seam cells (**f**). Each data point represents the mean of at least two replicates (each batch with at least 20 worms) and error bar represents the STD

Of the six DE miRNAs, five (*lin-4, miR-48, miR-84, miR-237*, and *miR-241*) were upregulated and one (*miR-246*) was downregulated (Fig. 3a, b). Notably, all of these except *miR-246* are known to be involved in both heterochronic development and a range of other processes [8, 9, 27, 28], a fact that was further supported by our tissue-enrichment analysis (Additional file 5: Figure S2). We next performed quantitative real-time PCR (qRT-PCR) validations which revealed similar, if comparatively lower (3–22-fold versus 2–12-fold in RNA-Seq and qRT-PCR, respectively), DE trends (Fig. 3B, C). Moreover, the *C. briggsae* orthologs of DE miRNAs were found to be likewise altered in *Cbr-pry-1(sy5353)* animals (Fig. 3d).

To elucidate whether *pry-1*-mediated miRNA regulation is stage-specific, we examined miRNA transcript levels by qRT-PCR in adult nematodes. While the expression of miRNAs was either unchanged or down-regulated in *pry-1(mu38)* (Additional file 6: Figure S3A), the pattern was different in *Cbr-pry-1(sy5353)* animals, i.e., three miRNA orthologs were up, two were down, and one was unchanged (Additional file 6: Figure S3B). We also used the existing *C. elegans miRNA::GFP* transgenic strains [29] to determine changes in miRNA expression in *pry-1(mu38)* animals and confirmed the qRT-PCR results (Additional file 7: Figure S4). Overall, the dissimilar expression trends of the analyzed miRNAs in adults from L1-stage nematodes suggests that the *pry-1*-miRNA network is likely temporally regulated.

*miR-246,* which a previous study showed to be involved in aging, oxidative stress, and thermo-sensation [30, 31], was the only miRNA to be downregulated in the *pry-1(mu38*) nematodes. Although no role in heterochronic development has yet been reported for *miR-246,* we herein demonstrate that *miR-246(n4636)* adults exhibit alae defects (Fig. 3e). Conversely, *scm::GFP* and *ajm-1::GFP* reporter-based expression analyses of the *miR-246(n4636)* adults did not reveal any significant change to the seam cells (Fig. 3f). This finding was supported by the results of the tissue-enrichment analysis (Additional file 5: Fig. S2). Interestingly, a hypodermal cell marker, *dpy-7::H1-wcherry*, revealed that the number of hypodermal cells was reduced in the mutant compared to control worms (45.1 ± 1.7, *n* = 20 and 51.9 ± 1.6, n = 20, respectively; also see Additional file 8: Figure S5). Further study is needed to determine the exact fate of these hypodermal cells in *miR-246* mutants.

### Many predicted gene targets of the mis-regulated heterochronic miRNAs are differentially expressed in the *pry-1(mu38)* mRNA transcriptome

**Fig. 4:**
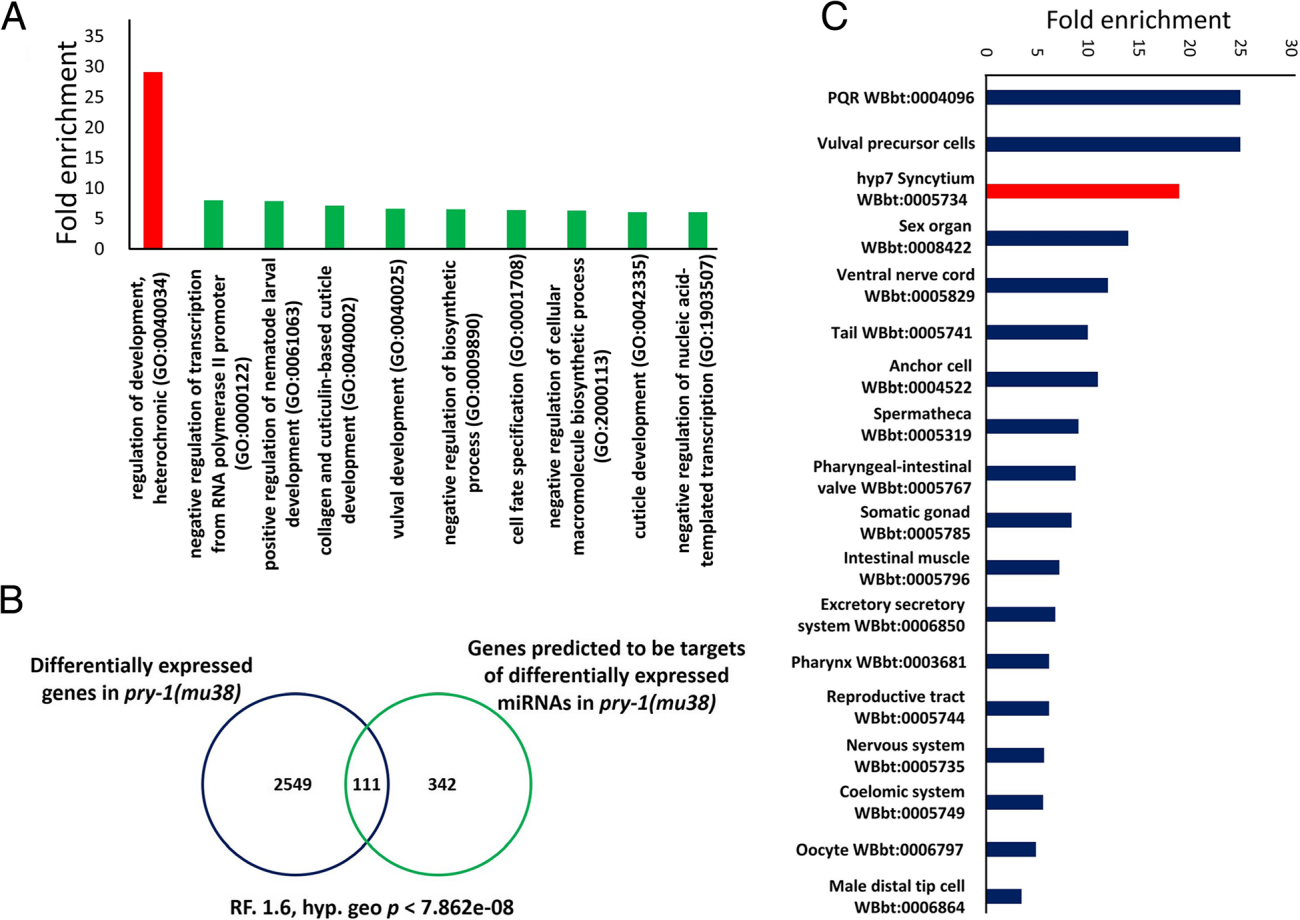
DE miRNAs and their predicted target genes in *pry-1(mu38)* are mostly linked to heterochronic development. (**a**) Enrichment analysis of potential targets of DE miRNAs using GO-terms associated with biological processes. A significant number of genes (29-fold enrichment) are linked to heterochronic development (colour coded in orange). (**b**) Venn diagram showing the overlap between previously reported protein-coding DE genes (2,660 genes, [25]) and predicted targets of DE miRNAs identified by Targetscan (435 genes, this study) in *pry-1(mu38)* animals. Further analysis revealed that 435 potential targets are shared between DE miRNAs (36 by *lin-4*, 115 by *miR-48*, 115 by *miR-84*, 36 by *miR-237*, 115 by *miR-241*, and 102 by *miR-246*). A total 111 genes are common between the two data sets, a number that is statistically significant based on the hypergeometric test. (**c**) Tissue-enrichment analysis of the common set of genes (111) revealed third highest fold enrichment in hypodermal syncytium cells (colour coded in orange)

miRNAs mediate the degradation or translational inhibition of their target mRNAs via binding between their seed sequence and an miRNA response element (MRE) in the 3′ untranslated region of their target. Therefore, we searched for miRNA targets using TargetScan online program (http://www.targetscan.org/vert_72/), and resultantly identified 453 unique targets. The gene ontology (GO) analysis revealed that these predicted miRNA targets were predominantly associated with processes such as the regulation of heterochrony (29-fold enrichment), the positive regulation of nematode larval development (8-fold enrichment), the molting cycle (5-fold enrichment), and collagen and cuticulin-based cuticle development (7-fold enrichment) (Fig. 4a).

A comparison of the predicted miRNA target genes with our recently published *pry-1(mu38)* mRNA transcriptome [25] revealed a significant overlap (111 genes, Representation factor: 1.6, hyp.geo *p* < 7.862 e-08) (Fig. 4b). Furthermore, a tissue-enrichment analysis showed that this subset of overlapping genes is frequently associated with the hypodermal syncytium (i.e. the third-most enriched subset) (Fig. 4c). Together, these findings suggest that PRY-1 is likely necessary for normal miRNA expression during seam cell development.

### Knockdowns of WNT asymmetric pathway components affect both the *pry-1(mu38)* phenotype and miRNA expression

**Fig. 5:**
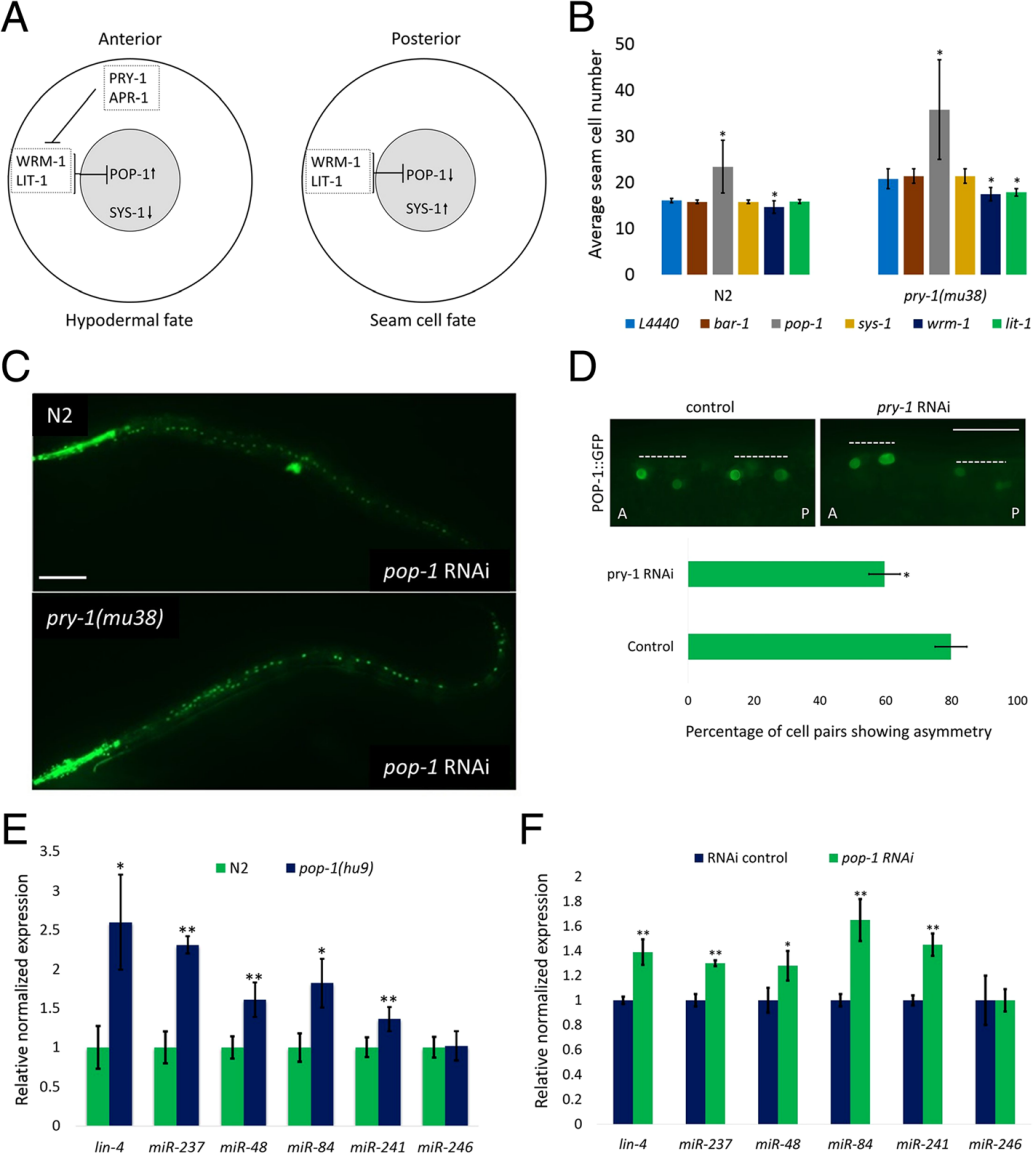
WNT asymmetry pathway components WRM-1, LIT-1, and POP-1 are important for PRY-1-mediated expression of miRNAs and seam cell development. (**a**) Schematic diagram showing the involvement of PRY-1 and other WNT asymmetry pathway components in the specification of seam cell fates. The levels of nuclear POP-1 are high in the anterior cell but low in the posterior cell. (**b**) RNAi knockdowns of *bar-1, pop-1, wrm-1, sys-1* and *lit-1* in control N2 and *pry-1(mu38)* animals. Each data point represents the mean of at least two batches (each batch with at least 30 worms) and error bar represents the STD. Student’s *t*-test was used to determine the statistical significance: *p < 0.05 (compared to L4440 control). (**c**) Representative images of control N2 and *pry-1(mu38)* animals following *pop-1* RNAi. The numbers of seam cells are increased in both cases. The phenotype is particularly enhanced in *pry-1* mutants (scale bar 0.1 mm). (**d**) Representative images of two adjacent POP-1::GFP fluorescing cell pairs from V1-V5 seam cell lineages following control and *pry-1* RNAi treatments (A-anterior, P- posterior, each dotted line marks a cell pair, scale bar 20 μm). POP-1 asymmetry is disrupted after *pry-1* RNAi, resulting in fewer cell pairs having asymmetric localisation of POP-1::GFP in their nuclei. (**e, f**) qRT-PCR analysis of miRNAs in *pop-1(hu9)* and *pop-1(RNAi)* worms at the L1 stage. Similar to *pry-1(mu38)*, all miRNAs, except *miR-246*, are overexpressed in both *pop-1(hu9)* (**e**) and *pop-1(RNAi)* (**f**) animals. Each data point represents the mean of two replicates and error bar represents the SEM, *p < 0.05, **p < 0.01

We induced RNAi-mediated silencing to examine interactions between *pry-1* and WNT asymmetry pathway components during seam cell development. The fates of seam cell daughters are specified by the nuclear levels of POP-1 that are high in the anterior cell (hypodermal fate) and low in the posterior cell (seam cell fate) [19, 32] (Fig. 5a). The results of our experiments revealed that knockdowns of *wrm-1* or *lit-1* suppresses the *pry-1(mu38)* seam cell phenotype (Fig. 5b), likely because PRY-1 promotes asymmetric division by negatively regulating both of these factors in anterior daughter cells (Fig. 5a). This is consistent with PRY-1 being localized to the anterior cortex of dividing seam cells [23]. A similar genetic interaction between *pry-1*, *wrm-1,* and *lit-1* was previously reported to occur during the asymmetric division of embryonic EMS cells [33].

In contrast to *wrm-1* and *lit-1*, *pop-1* RNAi exacerbated the *pry-1(mu38)* phenotype, resulting in a significant increase in the number of seam cells (35.9 ± 10.8 in *pry-1(mu38); pop-1(RNAi)*, compared to 20.8 ± 2.1 in *pry-1(mu38),* and 23.5 ± 5.7 in *pop-1(RNAi)*) (Fig. 5b, c). We also examined nuclear POP-1 asymmetry following RNAi knockdown of *pry-1* and found that it was disrupted (Fig. 5d). These results agree with nuclear POP-1 levels being differentially regulated by WRM-1 and LIT-1 to be higher in the anterior, and lower in the posterior daughter cell [19, 32] (Fig. 5a) Together, our findings support that *pop-1* likely limits the number of seam cells that are produced by promoting the asymmetric division of their precursors. Since the asymmetric localization of WRM-1, LIT-1, and POP-1 are known to mediate fate specification in presumptive seam cells, our results suggest that PRY-1 likely facilitates the maintenance of these asymmetric expression patterns.

Given that the nuclear POP-1 and SYS-1 ratio determines the fate of daughter cells and SYS-1 localization is disrupted in animals lacking PRY-1 function [34], we also examined the effect of *sys-1* knockdown. The results of this analysis showed no effect on seam cell division in *pry-1(mu38)* animals (Fig. 5b). We likewise knocked down another β-Catenin family member, *bar-1*, in the mutant strain and found that doing so did not alter the number of seam cells (Fig. 5b). Overall, the data support the possibility that β-Catenin family members are functionally redundant during seam cell division, consistent with previous studies [19], although do not exclude the possibility that PRY-1 role in seam cells is independent of BAR-1 and SYS-1.

To examine whether WRM-1, LIT-1, and POP-1 asymmetries affect miRNA expression during seam cell division, we next quantified miRNA levels in animals in which POP-1 function was compromised. As in *pry-1(mu38)* mutants, the expression levels of *lin-4, miR-48, miR-84*, *miR-237*, and *miR-241,* were found to be significantly upregulated in both *pop-1(hu9)* and *pop-1(RNAi)* worms; however, no change to *miR-246* expression was observed (Fig. 5e, f)*.* Furthermore, the bioinformatic analysis revealed multiple TCF/LEF consensus binding sites (SCTTTGATS; S = G/C) [35, 36] in the 5′ regulatory region of each of these miRNAs, except for *miR-246* where a single site is found near the transcriptional start site (See Methods, Additional file 9: Figure S6), suggesting that their transcription may be inhibited by POP-1. Together, these data allow us to conclude that miRNAs act downstream of POP-1, which agrees with a previous model [18], and also suggest that PRY-1 may interact with WRM-1, LIT-1, and POP-1 to negatively regulate the expression of heterochronic miRNAs during seam cell development.

### HBL-1 and LIN-28 act genetically downstream of PRY-1 and POP-1 signaling during asymmetric seam cell division

To further examine the role of *pry-1* in miRNA-mediated heterochronic development, we focused on three known miRNA targets, *hbl-1, lin-14*, and *lin-28*. Previous studies have shown that *lin-14* and *lin-28* are targeted by *lin-4* [3, 5, 37, 38], whereas *hbl-1* and *lin-28* are targeted by another *lin-4* family member *miR-237* [8, 39, 40] as well as *let-7* family members *miR-48*, *miR-84,* and *miR-241* [9, 17].

**Fig. 6:**
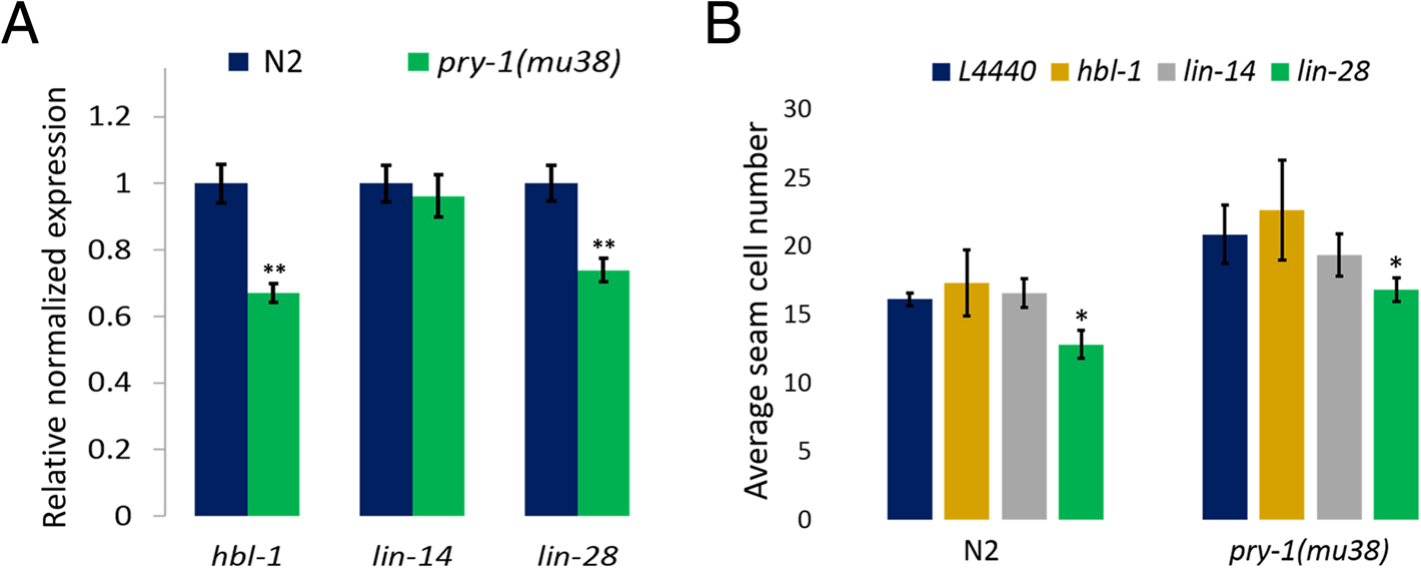
Analysis of heterochronic genes in *pry-1*-mediated seam cell development. (**a**) qRT-PCR experiments at the L1 stage showed that *hbl-1* and *lin-28* are significantly downregulated in *pry-1(mu38)* animals. Each data point represents the mean of two replicates and error bar represents the SEM, **p < 0.01. (**b**) RNAi KD of *lin-28* in *pry-1(mu38)* mutants rescued the seam cell defect. Each data point represents the mean of three batches (each batch with at least 30 worms) and error bar represents the STD. Student’s *t*-test was used to determine the statistical significance: *p < 0.05 (compared to L4440 control)

The qRT-PCR analysis showed that while *lin-14* expression levels were unchanged, *hbl-1* and *lin-28* were significantly downregulated in L1-stage *pry-1(mu38)* animals (Fig. 6a). Together with results described in previous sections, this observation allows us to propose that *pry-1* may function upstream of miRNAs to promote expression of *hbl-1* and *lin-28*. To further examine the regulatory network of *pry-1*, RNAi was carried out. The results revealed that while *lin-14* and *hbl-1* RNAi had no marked impact on seam cells, *lin-28* RNAi caused a significant reduction in the seam cell number in both *pry-1(mu38)* and control animals (Fig. 6b).

## Discussion

In this paper we describe a genetic pathway of PRY-1/Axin signaling in seam cell development. Using a combination of mutant analysis and reporter gene expression we show that PRY-1 is involved in L2-specific seam cell division. To identify the genes regulated by *pry-1*, we performed whole genome miRNA profiling at the late-L1 stage. The results revealed six DE miRNAs in *pry-1* mutants. Five of these, belonging to *lin-4* and *let-7* families (*lin-4*, *miR-48, miR-84*, *miR-237,* and *miR-241*), were upregulated whereas *miR-246* was the only miRNA that was downregulated. A similar trend was also observed in *C. briggsae pry-1* mutants suggesting that *pry-1* plays a conserved role in miRNA regulation in *Caenorhabditis* nematodes.

Three of the overexpressed miRNAs in *pry-1* mutants, namely *miR-48, miR-84*, and *miR-241* (*let-7* family members), are known to redundantly control the L2-L3 larval-stage transition [9]. While *C. elegans* nematodes carrying a mutation in any one of these three miRNAs have been shown to exhibit a normal phenotype*, miR-48*/*miR-84* double mutants display retarded molting and a higher number of seam cells as a result of reiterated symmetric divisions during the L2 stage [9]. This seam cell phenotype is further exacerbated in *miR-48/miR-84/miR-241* triple mutants [9], while conversely, *miR-48-*overexpression mutants were shown to exhibit a reduced number of seam cells due to ‘skipping’ of L2-specific symmetric divisions [41]. The other two miRNAs upregulated in the *pry-1(mu38)* mutants are *lin-4* and *miR-237 (lin-4* family members [42]). A previous study showed that, although a *miR-237*-knock-out mutant does not directly incur a heterochronic defect, it enhances the seam cell phenotype exhibited by *lin-4(e912)*; *lin-14(n179ts)* double mutant animals [8].

Unlike *lin-4* and *let-7* family of DE miRNAs, *miR-246* is not known to play a role in heterochronic development although it is involved in other processes [30, 31]. Consistent with previous studies, our analysis of *miR-246* mutants did not reveal any changes in the number of seam cells. Thus, *pry-1*-mediated *miR-246* regulation may participate in other biological events.

The fact that all but one DE miRNAs in *pry-1* mutants are involved in heterochronic pathway suggests an important role of *pry-1* in this developmental process. This was further strengthened by our data showing a high enrichment for miRNA predicted target genes associated with GO-term processes such as regulation of heterochrony. Moreover, we observed a significant overlap between DE miRNA predicted targets and *pry-1(mu38)* mRNA transcriptome [25] that included many genes expressed in hypodermal syncytium.

To understand the regulation of miRNAs by *pry-1* we studied the involvement of WNT asymmetry pathway components using an RNAi approach. It was shown earlier that in the absence of PRY-1, localizations of WRM-1, LIT-1, and SYS-1 are disrupted [23, 34]. As expected, the examination of seam cell phenotypes in *pry-1(mu38)* animals following RNAi knockdowns of these genes revealed that *wrm-1* and *lit-1* are necessary for *pry-1* function. Thus, PRY-1 may affect seam cell number by localizing in the anterior cortex of dividing seam cells [23] and thereby lowering WRM-1 and LIT-1-mediated nuclear POP-1 levels in anterior daughters. This plausible explanation is supported by our finding that POP-1 asymmetry is affected in animals with reduced PRY-1 function. To test this further, we examined miRNA expression in *pop-1(hu9)* and *pop-1(RNAi)* worms. As expected, all five (*lin-4, miR-48, miR-84, miR-237,* and *miR-241*) were found to be overexpressed. Moreover, multiple TCF/LEF binding sites were detected in the 5′ regulatory regions of miRNAs. Together, these findings raise the possibility of POP-1 acting as a transcriptional regulator of miRNAs.

**Fig. 7:**
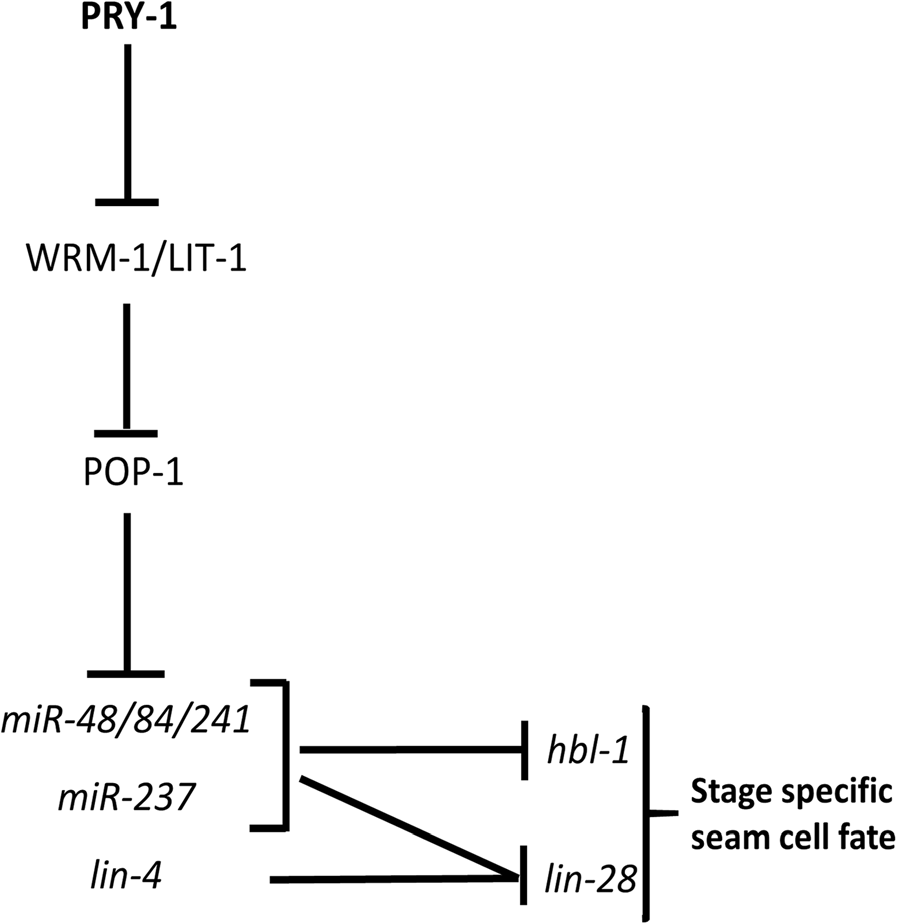
A model summarizing genetic interactions between PRY-1, WNT asymmetric pathway components (WRM-1, LIT-1, and POP-1), heterochronic miRNAs, and the targets of heterochronic miRNAs during L2 stage seam cell development. Our data supports the Ren and Zhang model [18] and places the WNT asymmetric pathway upstream of miRNAs

We thus propose a model summarizing our findings (Fig. 7), in which PRY-1 acts upstream of WRM-1, LIT-1, POP-1, and DE miRNAs (except *miR-246*). Since the miRNAs regulate the expression of protein-coding genes during heterochronic development, we tested three of their known targets *hbl-1, lin-14,* and *lin-28*. The results of our experiments suggested that *hbl-1* and *lin-28* act downstream of *pry-1*; however, only *lin-28* appears to function in *pry-1*-mediated asymmetric cell division. The above genetic interactions are consistent with earlier findings where *lin-28* was shown to act downstream of the WNT asymmetry pathway components and included in a network consisting of *lin-4* and *let-7*-family of miRNAs and their targets during seam cell development [17, 18]. Our model is unique in that it places PRY-1 upstream of WRM-1, LIT-1, and POP-1-mediated miRNA transcriptional network.

## Conclusions

Overall, the data presented in this paper demonstrate the important role of PRY-1/Axin in the regulation of miRNAs and their heterochronic gene targets in a pathway that involves WNT asymmetry pathway components WRM-1, LIT-1, and POP-1 during seam cell development. Furthermore, since seam cell defects are also exhibited by *C. briggsae pry*-*1* mutants, and that *Cbr-pry-1* is necessary for the normal expression of these miRNA orthologs, our work has revealed that the role of *pry-1* in seam cell development is conserved amongst nematodes.

## Methods

### Strains, culture condition, and RNAi

Nematodes were grown on standard NG-agar culture plates seeded with *E. coli* bacteria (OP50) [43]. Cultures were maintained at 20 °C. Strains used in the study are listed in a supplementary table (Additional file 10: Table S4). RNAi-mediated gene silencing was performed using a protocol previously published by our laboratory [24].

### Microscopy

Nematodes were mounted on a glass slide containing 2% agarose and 0.02 M NaN_3_ and observed using an Axiovision Zeiss microscope. Seam cell nuclei were counted, and adult lateral alae were scored using Nomarski differential interference contrast and epifluorescence optics. Images were acquired using NIS Element software (Nikon, USA) with a Hamamastu Camera that was mounted on a Nikon 80i upright microscope.

### Analysis of seam cell division

The fates of daughter seam cells were determined in *C. elegans* using *scm::GFP* and *ajm-1::GFP* markers, and in *C. briggsae* using the seam cell adherens-junction marker, *Cel-dlg-1::GFP*. After 8 h of feeding, nematodes expressing GFP that had completed the first larval seam cell division, and that had 10 seam cells per side, were chosen for analysis. Seam cell divisions were monitored at approximately 6 h intervals until the late L4 stage, when divisions ceased.

### Cuticle integrity assay

A solution containing 1% hypochlorite and 0.25 M NaOH was prepared as previously reported [44], and aliquoted (500 μl) into a 48-well plate. Individual gravid adult worms were transferred to each well, and the plate was agitated at 30 s intervals. The time to the first major cuticle break was recorded by direct observation using a dissecting stereoscope (SMZ 645; Nikon Corporation, Japan).

### Molecular biology and bioinformatics

For qRT-PCR experiments, synchronous cultures were prepared by bleaching gravid hermaphrodites as described previously [45] except that eggs were allowed to hatch on the NG-agar plates. The bleaching process was repeated. The eggs were finally transferred onto plates and grown till the desired stage. Because *pry-1* mutants grow slower than controls, RNA was extracted from age-matched animals. The L1 larvae were grown till 16 h (N2/AF16) and 18 h (*pry-1* mutants) whereas adults were incubated for 52 h (N2/AF16) and 58 h (*pry-1* mutant). Total RNA was extracted from animals using the trizol reagent (Catalog Number T9424, Sigma-Aldrich, Canada), according to the manufacturer’s instructions. cDNAs for protein-encoding genes and miRNAs were synthesized using oligo-dT and specific stem-loop primers, respectively (Additional file 11: Table S5), and by using the qScript cDNA synthesis kit (Catalog Number 95047–025, Quantabio, Canada) according to manufacturer’s instructions.

qRT-PCR was performed (in triplicate) in the Bio-Rad cycler CFX 96 using appropriate primers (Additional file 11: Table S5), and SensiFAST SYBR Green Kit (Catalog Number BIO-98005, BIOLINE, USA), according to the manufacturer’s instructions. The expression levels of miRNAs and protein-coding genes were normalized to those of *miR-2* and *pmp-3,* respectively. Ct and *p* values were calculated using CFX manager software (Bio-Rad, Canada).

A *lin-28* RNAi plasmid was constructed by inserting 2949 bp of the *lin-28* coding sequence into the L4440 vector. A DNA fragment was obtained via PCR using the listed primers (Additional file 11: Table S5), under the specified PCR conditions.

To identify TCF/LEF family binding sites in the 5′ upstream genomic region of the miRNAs, MatInspector software (https://www.genomatix.de/) was used with default settings.

### RNA-Seq experiment

The steps for miRNA RNA-Seq in *pry-1(mu38)* mutants were similar to mRNA RNA-Seq that we reported earlier [25]. The *pry-1* miRNA transcriptome profile can be found in the GEO archive with accession number GSE130039. Synchronized L1 worms were harvested by two successive bleaching to obtain a homogenous population and total RNA was isolated. Small RNA sequencing libraries were prepared, and samples were analysed using the Genome Analyzer IIx platform (Illumina Inc., USA) at the McGill University Genome Quebec sequencing facility.

A total of 36,656,022 reads of small RNAs (15–25 nt) were generated from the four samples of *C. elegans* examined, of which 10,453,527 sequences aligned perfectly to the *C. elegans* genome. Overall, we detected perfect matches to the precursor forms of 161 out of the 250 miRNAs annotated in miRBase (http://www.mirbase.org) WBcel235 for *C. elegans*. DE analysis of the known miRNAs led to identification of eight miRNAs that were altered in *pry-1* mutants, of which two (*miR-353* and *miR-2208a*) were excluded due to false predictions (Additional file 2: Table S1).

Although previous studies have reported miRNAs in the *C. elegans* genome (e.g., see [42, 46], due to increased depth of our sequencing data we expected to uncover additional new candidates. After eliminating rRNA, tRNA, and ncRNA reads, the remaining unannotated reads were processed for novel miRNA discovery, as discussed below. We focused on the 30,300 non-redundant un-annotated reads that aligned to the *C. elegans* genome in control N2 and *pry-1(mu38)* animals. To discover novel miRNAs, the miRNA discovery package miRDeep2 (https://www.mdc-berlin.de/n-rajewsky#t-data,software&resources) was used. The analysis predicted a total of 243 miRNAs using read count cut-off of 5-fold (or 187 with cut-off of 10-fold) (Additional file 3: Table S2). We then used an additional criterion to further examine these candidates, i.e., a higher miRDeep Score (> 10). This led to the identification of 64 novel miRNAs at the 5-fold read count cut-off (or 61 when the read count was set at 10-fold) (Additional file 3-4: Table S2, S3**)**. The authenticity of these novel miRNAs was tested by RNAfold, which confirmed that these produce miRNA stem loop structure [47].

### Statistical analyses

Statistics were performed by two-tailed student’s *t* test after testing for equal distribution of the data and equal variances within the data set. The *p* values of 0.05 and less were considered statistically significant. The data are presented as either mean ± standard deviation of the mean (STD) or mean ± standard error of mean (SEM). Graphs were prepared using Microsoft Excel. Hypergeometric probability related tests were done using an online program (http://nemates.org/MA/progs/overlap_stats.html).

## Additional files

Additional file 1: **Figure S1.** *C. elegans pry-1* open reading frame showing the region affected by *gk3682* mutation. The exons and introns are indicated by boxes and lines, respectively. The translational start and stop sites are marked. The sequence deleted in *gk3682* allele (738 bp) is shown by a rectangle. As part of the CRISPR editing process, the excised portion is replaced by a 5419 bp *myo-2::GFP* containing cassette. The allele and sequencing data were kindly provided by Dr. Moerman’s lab. (TIF 157 kb)

Additional file 2: **Table S1.** A list of miRNAs in *pry-1(mu38)* animals. The file contains two spreadsheets that list all miRNAs identified by RNA-Seq experiment (used for volcano plot, see Fig. 3a) and DE miRNAs. (XLSX 25 kb)

Additional file 3: **Table S2.** Total number of novel miRNAs in *C. elegans*. The table shows the number of predicated miRNAs based on different miRDeep scores and read count cut-offs. (DOCX 13 kb)

Additional file 4: **Table S3.** A list of novel miRNAs in *C. elegans*. The IDs of miRNAs are based on the sequential numbering of unique reads in our analysis. For each novel miRNA, the table lists the miRDeep score, read count, chromosomal location, and the sequence. (XLSX 380 kb)

Additional file 5: **Figure S2.** Tissue-enrichment analysis of DE miRNAs. The analysis was done using the miRNA discovery tool miRDeep2 (see the RNA-Seq section in Methods). For each miRNA, colored areas represent tissue-specific expression. (TIF 9730 kb)

Additional file 6: **Figure S3.** Adult stage qRT-PCR analysis of heterochronic miRNAs in *C. elegans* and *C. briggsae pry-1* mutants. (A) *pry-1(mu38)* adults show differences in the pattern of miRNA expression compared to the L1 stage. All miRNAs, except *lin-4* and *miR-48,* are downregulated. (B) *Cbr-pry-1(sy5353)* adults show altered expression of *miR-246*, *miR-48* and *miR-84*. Each data point represents the mean of two replicates and error bar represents the SEM, *p < 0.05, **p < 0.01 (TIF 3950 kb)

Additional file 7: **Figure S4.** miRNA expression analysis in *pry-1(mu38)* adults using a *GFP* reporter. (A) Representative images of *PmiR-48::GFP, PmiR-84::GFP, PmiR-241::GFP,* and *PmiR-246::GFP* reporters in control N2 and *pry-1(mu38)* animals. The scale bar is 0.1 mm. (B) Quantification of fluorescence intensity using an arbitrary unit (a.u.) scale. Each data point represents the mean of two replicates (at least 20 animals each) and error bar represents the STD. Student’s *t*-test was used to determine the statistical significance: *p < 0.05. (TIF 7910 kb)

Additional file 8: **Figure S5.** Representative images of control N2 and *miR-246(n4636)* mutants showing hypodermal cells (based on *dpy-7::H1-wcherry* reporter). The mutant animal shows fewer hypodermal cells. (TIF 9910 kb)

Additional file 9: **Figure S6.** Line drawings of TCF/LEF putative binding sites in the 5′ upstream regions of miRNA genes (within 1500 bp of transcriptional start site). Each putative binding site is shown by a coloured square box. The numbers in brackets next to boxes show matrix similarity scores. (TIF 7130 kb)

Additional file 10: **Table S4.** A list of strains used in this study. (DOCX 33 kb)

Additional file 11: **Table S5.** A list of primers used in this study. (DOCX 16 kb)

## Data Availability

The datasets supporting the conclusions of this article are provided in figures, tables, and additional files. miRNA transcriptome data of *pry-1* mutants can be found in the NCBI GEO archive database with accession number GSE130039.

## Abbreviations

DE: Differentially expressed
GO: Gene ontology
miRNAs: microRNAs
MRE: miRNA response element
NLK: NEMO-like kinase
qRT-PCR: quantitative real-time PCR
SEM: Standard error of the mean
STD: Standard deviation
TCF/LEF: T-cell factor/lymphoid enhancer factor
