# Supplementary figures and images for: Role of PRY-1/Axin in heterochronic miRNA-mediated seam cell development
Source: BMC Dev Biol. 2019 Jul 15;19:17. doi: 10.1186/s12861-019-0197-5 (PMC6631683; doi:10.1186/s12861-019-0197-5)

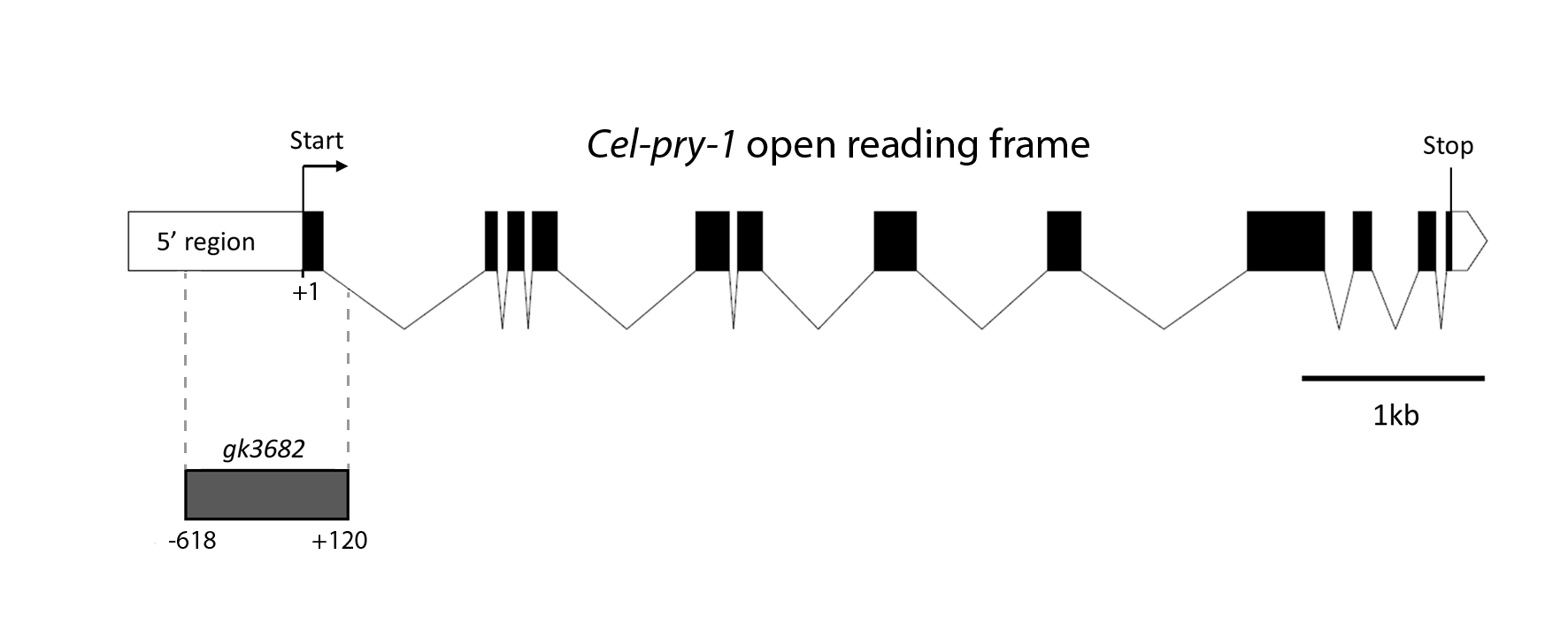

Supplement: Supplementary file 1 — Figure S1. C. elegans pry-1 open reading frame showing the region affected by gk3682 mutation. The exons and introns are indicated by boxes and lines, respectively. The translational start and stop sites are marked. The sequence deleted in gk3682 allele (738 bp) is shown by a rectangle. As part of the CRISPR editing process, the excised portion is replaced by a 5419 bp myo-2::GFP containing cassette. The allele and sequencing data were kindly provided by Dr. Moerman’s lab. (TIF 157 kb) [file 12861_2019_197_MOESM1_ESM.tif]

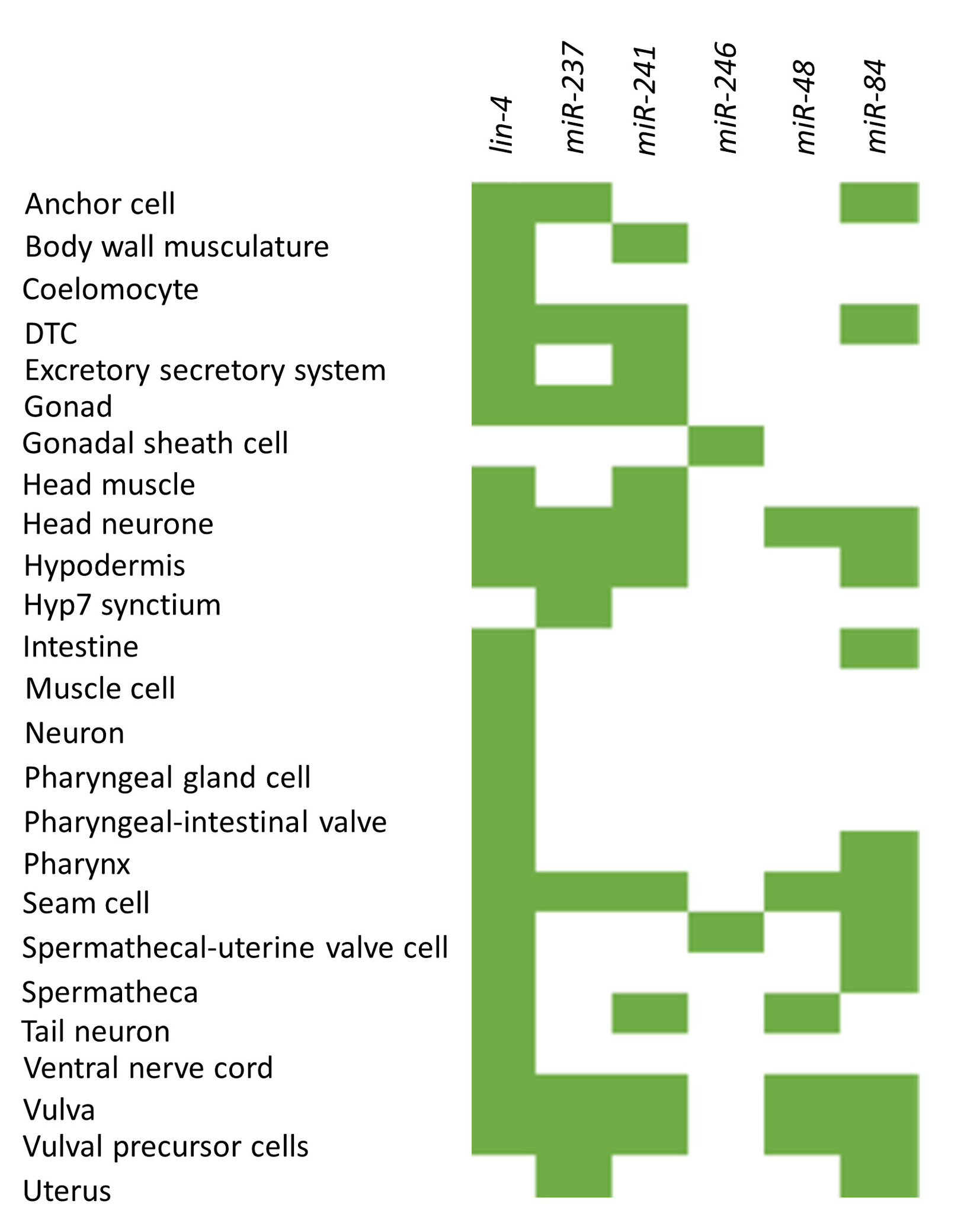

Supplement: Supplementary file 5 — Figure S2. Tissue-enrichment analysis of DE miRNAs. The analysis was done using the miRNA discovery tool miRDeep2 (see the RNA-Seq section in Methods). For each miRNA, colored areas represent tissue-specific expression. (TIF 9730 kb) [file 12861_2019_197_MOESM5_ESM.tif]

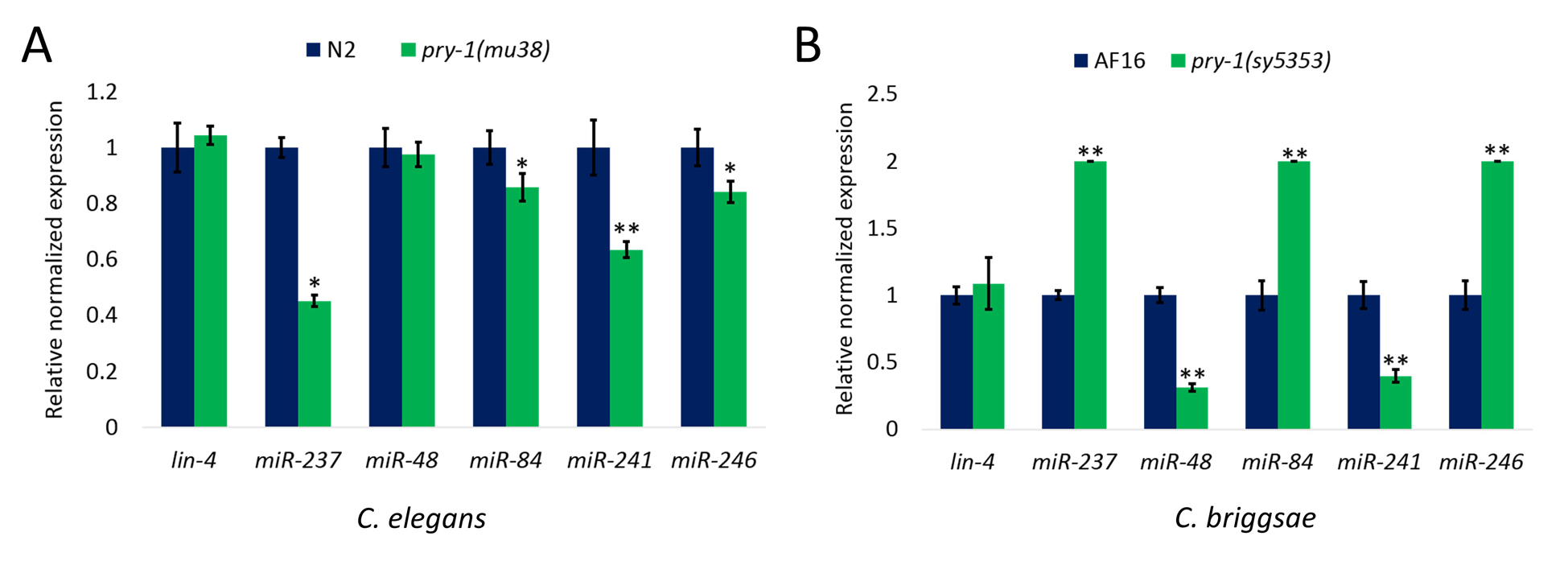

Supplement: Supplementary file 6 — Figure S3. Adult stage qRT-PCR analysis of heterochronic miRNAs in C. elegans and C. briggsae pry-1 mutants. (A) pry-1(mu38) adults show differences in the pattern of miRNA expression compared to the L1 stage. All miRNAs, except lin-4 and miR-48, are downregulated. (B) Cbr-pry-1(sy5353) adults show altered expression of miR-246, miR-48 and miR-84. Each data point represents the mean of two replicates and error bar represents the SEM, *p < 0.05, **p < 0.01 (TIF 3950 kb) [file 12861_2019_197_MOESM6_ESM.tif]

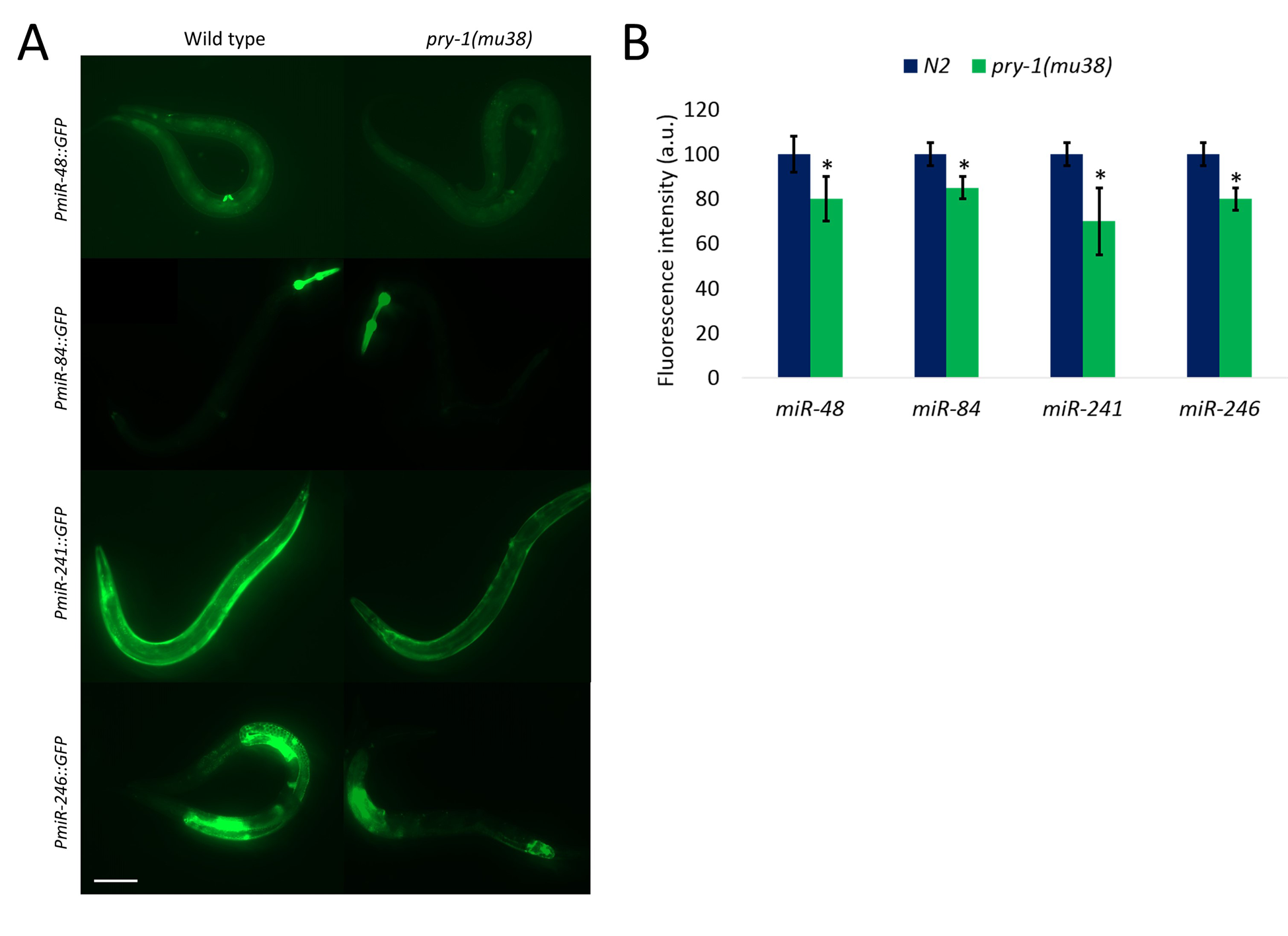

Supplement: Supplementary file 7 — Figure S4. miRNA expression analysis in pry-1(mu38) adults using a GFP reporter. (A) Representative images of PmiR-48::GFP, PmiR-84::GFP, PmiR-241::GFP, and PmiR-246::GFP reporters in control N2 and pry-1(mu38) animals. The scale bar is 0.1 mm. (B) Quantification of fluorescence intensity using an arbitrary unit (a.u.) scale. Each data point represents the mean of two replicates (at least 20 animals each) and error bar represents the STD. Student’s t-test was used to determine the statistical significance: *p < 0.05. (TIF 7910 kb) [file 12861_2019_197_MOESM7_ESM.tif]

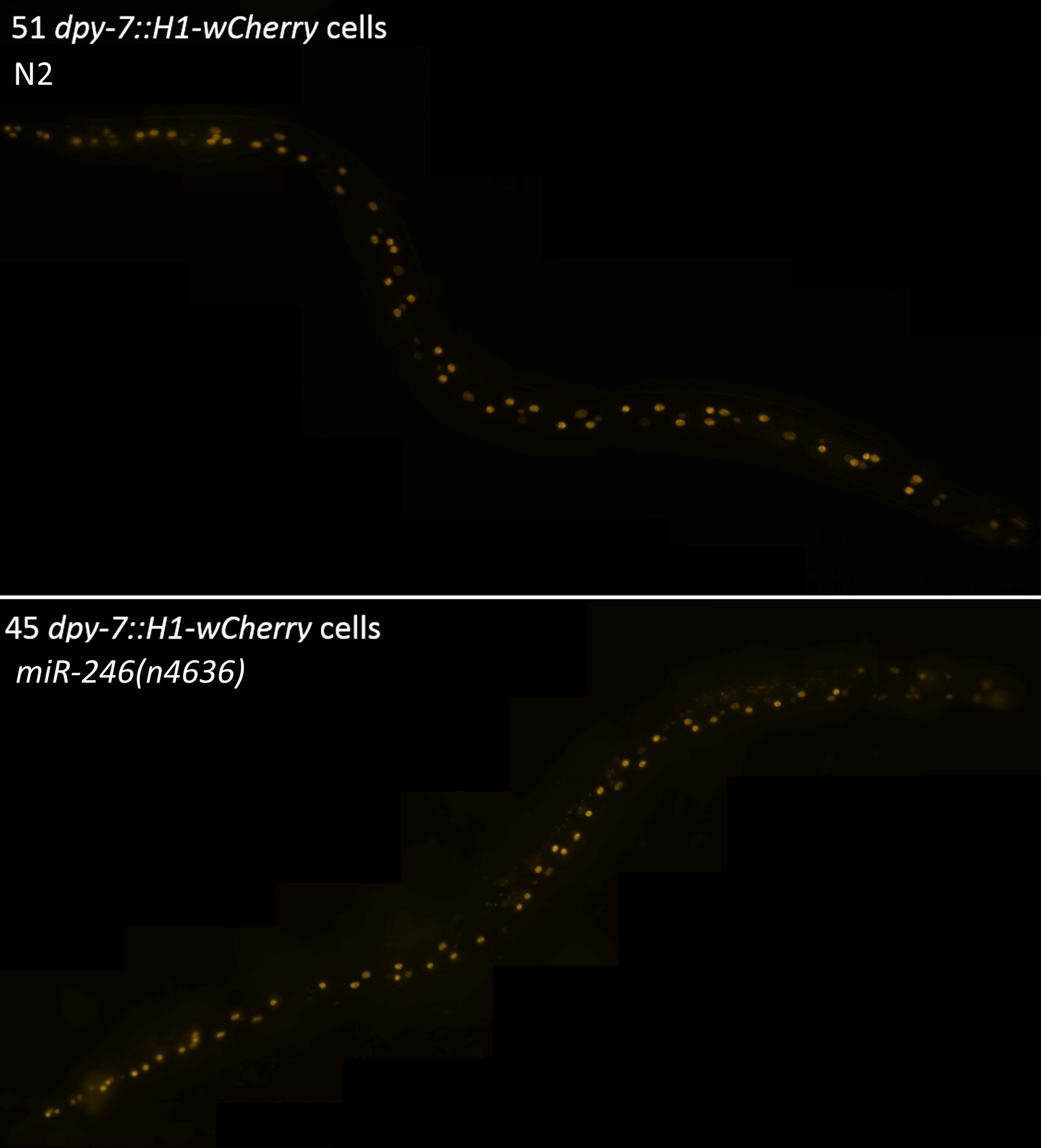

Supplement: Supplementary file 8 — Figure S5. Representative images of control N2 and miR-246(n4636) mutants showing hypodermal cells (based on dpy-7::H1-wcherry reporter). The mutant animal shows fewer hypodermal cells. (TIF 9910 kb) [file 12861_2019_197_MOESM8_ESM.tif]

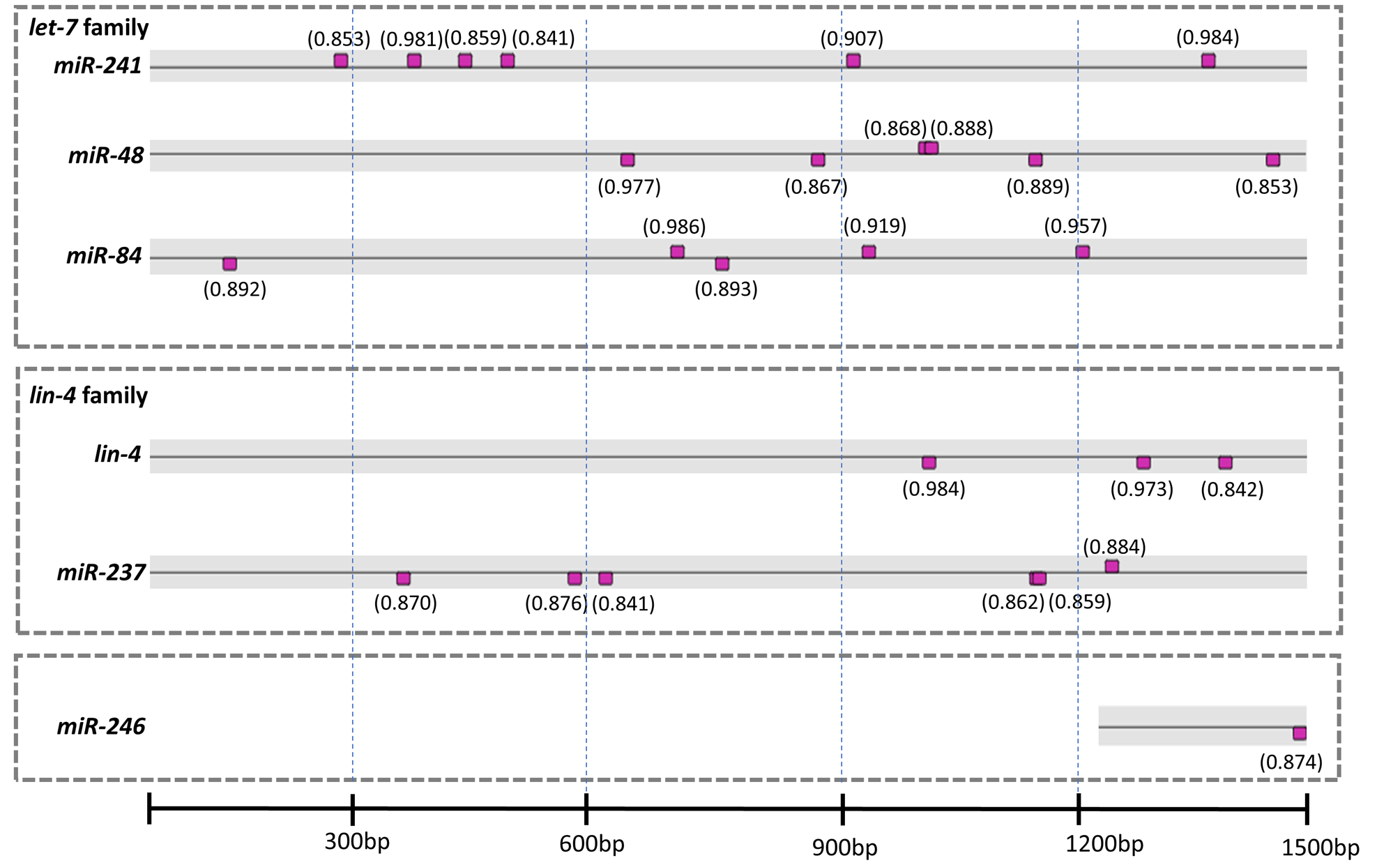

Supplement: Supplementary file 9 — Figure S6. Line drawings of TCF/LEF putative binding sites in the 5′ upstream regions of miRNA genes (within 1500 bp of transcriptional start site). Each putative binding site is shown by a coloured square box. The numbers in brackets next to boxes show matrix similarity scores. (TIF 7130 kb) [file 12861_2019_197_MOESM9_ESM.tif]
