# Supplementary material for: Role of PRY-1/Axin in heterochronic miRNA-mediated seam cell development
Source: BMC Dev Biol. 2019 Jul 15;19:17. doi: 10.1186/s12861-019-0197-5 (PMC6631683; doi:10.1186/s12861-019-0197-5)
Supplement: Supplementary file 3 — Table S2. Total number of novel miRNAs in C. elegans. The table shows the number of predicated miRNAs based on different miRDeep scores and read count cut-offs. (DOCX 13 kb) [file 12861_2019_197_MOESM3_ESM.docx]

**Additional file 3: Table S2: Total number of Novel miRNAs in *C. elegans*.**

|  | | Number of novel miRNA genes | |
| --- | --- | --- | --- |
|  | mirDeep Score | Read count (> 10-fold) | Read count (> 5-fold) |
| *C. elegans* | 10 | 61 | 64 |
|  | 1 | 187 | 243 |
