# Supplementary material for: Role of PRY-1/Axin in heterochronic miRNA-mediated seam cell development
Source: BMC Dev Biol. 2019 Jul 15;19:17. doi: 10.1186/s12861-019-0197-5 (PMC6631683; doi:10.1186/s12861-019-0197-5)
Supplement: Supplementary file 10 — Table S4. A list of strains used in this study. (DOCX 33 kb) [file 12861_2019_197_MOESM10_ESM.docx]

**Additional file 10: Table S4:** List of strains used in this study.

| **Strain** | **Genotype** | **Reference** |
| --- | --- | --- |
| N2 | *C. elegans* reference strain |  |
| AF16 | *C. briggsae* reference strain |  |
| DY220 | *pry -1(mu38) I* | [1] |
| DY250 | *Cbr*-*pry-1(sy5353) I* | [2] |
| JU1078 | *mfEx33[myo-2::RFP + Cel-dlg-1::GFP]* | [2], M.-A. Felix lab |
| DY319 | *Cbr*-*pry-1(sy5353) I*; *mfEx[myo-2::RFP + Cel-dlg-1::GFP]* | [2] |
| DY629 | *pry-1(mu38) I*; *wIs78[scm::GFP+ajm-1::GFP+F58E10 (cosmid)+unc-119(+)] IV* | This study |
| DY630 | *pry-1(mu38) I*; *maIs150[mir-48p::GFP+unc-119(+)]* | This study |
| DY631 | *pry-1(mu38) I*; *maIs140[mir-241p::GFP+unc-119(+)]* | This study |
| DY636 | *pry-1(mu38) I*; *maIs138[mir-84p::GFP+unc-119(+)]* | This study |
| DY637 | *pry-1(mu38) I; maIs236[mir-246p::GFP+unc-119(+)]* | This study |
| DY638 | *pry-1(gk3682)I*;*wIs78[scm::GFP+ajm-1::GFP+F58E10 (cosmid)+unc-119(+)] IV* | This study |
| DY646 | *miR-246(n4636) IV; stIs11597[dpy-7::H1-wCherry+unc-119(+)]* | This study |
| DY649 | *miR-246(n4636) IV; wIs51 [scm::GFP+unc-119(+)] V* | This study |
| JR667 | *unc-119(e2498::Tc1) III*; *wIs51 [scm::GFP+unc-119(+)] V* | [3] |
| JK3437 | *him-5(e1490) V, qIs74 [sys-1p::GFP::pop-1 + unc-119(+)]* | [4] |
| KN562 | *pop-1(hu9) I* | [1] |
| MT15020 | *miR-246(n4636) IV* | [5] |
| RG733 | *wIs78[scm::GFP+ajm-1::GFP+F58E10 (cosmid)+unc-119(+)] IV* | [3] |
| RW11597 | *unc-119(tm4063) III; stIs11597[dpy-7::H1-wCherry+unc-119(+)]* | R. H. Waterston lab |
| VT1259 | *unc-119(ed3) III; maIs150[mir-48p::GFP+unc-119(+)]* | [6] |
| VT1607 | *unc-119(ed3) III; maIs236[mir-246p::GFP+unc-119(+)]* | [6] |
| VT1189 | *unc-119(ed3) III; maIs140[mir-241p::GFP+unc-119(+)]* | [6] |
| VT1160 | *unc-119(ed3) III; maIs138[mir-84p::GFP+unc-119(+)]* | [6] |
| VC3710 | *pry-1(gk3682) I* | This study |

1. Korswagen HC, Coudreuse DY, Betist MC, van de Water S, Zivkovic D, Clevers HC: **The Axin-like protein PRY-1 is a negative regulator of a canonical Wnt pathway in *C. elegans***. *Genes Dev* 2002, **16**(10):1291-1302.

2. Seetharaman A, Cumbo P, Bojanala N, Gupta BP: **Conserved mechanism of Wnt signaling function in the specification of vulval precursor fates in C. elegans and C. briggsae**. *Dev Biol* 2010, **346**(1):128-139.

3. Koh K, Rothman JH: **ELT-5 and ELT-6 are required continuously to regulate epidermal seam cell differentiation and cell fusion in C. elegans**. *Development* 2001, **128**(15):2867-2880.

4. Siegfried KR, Kidd AR, 3rd, Chesney MA, Kimble J: **The sys-1 and sys-3 genes cooperate with Wnt signaling to establish the proximal-distal axis of the Caenorhabditis elegans gonad**. *Genetics* 2004, **166**(1):171-186.

5. Miska EA, Alvarez-Saavedra E, Abbott AL, Lau NC, Hellman AB, McGonagle SM, Bartel DP, Ambros VR, Horvitz HR: **Most Caenorhabditis elegans microRNAs are individually not essential for development or viability**. *PLoS Genet* 2007, **3**(12):e215.

6. Martinez NJ, Ow MC, Reece-Hoyes JS, Barrasa MI, Ambros VR, Walhout AJ: **Genome-scale spatiotemporal analysis of Caenorhabditis elegans microRNA promoter activity**. *Genome Res* 2008, **18**(12):2005-2015.
