# Supplementary material for: Role of PRY-1/Axin in heterochronic miRNA-mediated seam cell development
Source: BMC Dev Biol. 2019 Jul 15;19:17. doi: 10.1186/s12861-019-0197-5 (PMC6631683; doi:10.1186/s12861-019-0197-5)
Supplement: Supplementary file 11 — Table S5. A list of primers used in this study. (DOCX 16 kb) [file 12861_2019_197_MOESM11_ESM.docx]

**Additional file 11: Table S5: List of primers used.**

| **Gene** | **GL #** | **Orientation** | **Sequence (5’-3’)** |
| --- | --- | --- | --- |
| *miR-2-5p* | 1247 | Forward | CTCAACTGGTGTCGTGGAGTCGGCAATTCAGTTGAGGCACATCA |
| *lin-4-5p* | 1331 | Forward | CTCAACTGGTGTCGTGGAGTCGGCAATTCAGTTGAGTCACACTT |
| *miR-48-5p* | 1245 | Forward | CTCAACTGGTGTCGTGGAGTCGGCAATTCAGTTGAGTCGCATCT |
| *miR-84-5p* | 1246 | Forward | CTCAACTGGTGTCGTGGAGTCGGCAATTCAGTTGAGTCTACAAT |
| *miR-237-5p* | 1355 | Forward | CTCAACTGGTGTCGTGGAGTCGGCAATTCAGTTGAGAGCTGTTC |
| *miR-241-5p* | 1332 | Forward | CTCAACTGGTGTCGTGGAGTCGGCAATTCAGTTGAGTCATTTCT |
| *miR-246-3p* | 1244 | Forward | CTCAACTGGTGTCGTGGAGTCGGCAATTCAGTTGAGGCTCCTAC |
| *Cbr-miR-84-5p* | 1255 | Forward | CTCAACTGGTGTCGTGGAGTCGGCAATTCAGTTGAGGACAGCAT |
| *Cbr-miR-237-5p* | 1357 | Forward | CTCAACTGGTGTCGTGGAGTCGGCAATTCAGTTGAGAGATGTCG |
| *miR-2-5p* | 1252 | Forward | ACACTCCAGCTGGGTATCACAGCCAGCTTT |
| *lin-4-5p* | 1333 | Forward | ACACTCCAGCTGGGTCCCTGAGACCTCAAG |
| *miR-48-5p* | 1250 | Forward | ACACTCCAGCTGGGTGAGGTAGGCTCAGTA |
| *miR-84-5p* | 1251 | Forward | ACACTCCAGCTGGGTGAGGTAGTATGTAAT |
| *miR-237-5p* | 1356 | Forward | ACACTCCAGCTGGGTCCCTGAGAATTCTCG |
| *miR-241-5p* | 1334 | Forward | ACACTCCAGCTGGGTGAGGTAGGTGCGAGA |
| *miR-246-3p* | 1249 | Forward | ACACTCCAGCTGGGTTACATGTTTCGGGTA |
| *Cbr-miR-84-5p* | 1256 | Forward | ACACTCCAGCTGGGTGAGGTAGTTTGCAAT |
| *Cbr-miR-237-5p* | 1358 | Forward | ACACTCCAGCTGGGTCCCTGAGAATGCTCC |
| *Cbr-miR-246-3p* | 1257 | Forward | ACACTCCAGCTGGGTTACATGTATTGGGTA |
| *Universal miRNA primer* | 1254 | Reverse | CTCAACTGGTGTCGTGGAGTCGGCAA |
| *pmp-3* | 747 | Forward | CTTAGAGTCAAGGGTCGCAGTGGAG |
|  | 748 | Reverse | ACTGTATCGGCACCAAGGAAACTGG |
| *hbl-1* | 1258 | Forward | TTGGCACAAAGAGCAAAGCC |
|  | 1259 | Reverse | GGCCATTCTGATCCTATTAAAGGTG |
| *lin-14* | 1262 | Forward | TGCGAGGAATGGGGAAATGG |
|  | 1263 | Reverse | GTCGAAGATCGGTTACTTCTTTCC |
| *lin-28* | 1264 | Forward | AAGTTGAAGATAGGCTGCCAGA |
|  | 1265 | Reverse | GTGTTGGTGACGGGAGCC |
| *miR-246* | 1325 | Forward | CGTCGGAAGATTCACTCCTG |
|  | 1326 | Reverse | CCTCACTGGTCATACTTCCC |
| *pry-1(gk3682)* | 1307 | Forward | CATCGCCTTCTATCGCCTTCTTGA |
|  | 1308 | Reverse | GCGAAGAACAAGTCGAGGTACTG |
| *lin-28 RNAi plasmid* | 1359 | Forward | TAAG**GGTACC**GTAGTATCGGAGGGAAGGAATG |
|  | 1360 | Reverse | TAAG**GCTAGC**GGTGGTAGTATGGTTTAGAGGG |
